# Femoral malalignment after gamma nail insertion in the lateral decubitus position

**DOI:** 10.1051/sicotj/2018033

**Published:** 2018-07-30

**Authors:** Hossam M.A. Abubeih, Osama Farouk, Mohammad Kamal Abdelnasser, Amr Atef Eisa, Galal Zaki Said, Wael El-adly

**Affiliations:** Orthopaedic Department, Assiut University, Assiut Egypt

**Keywords:** Proximal femoral fractures, Gamma nail, Femoral angular malalignment, Femoral rotational malalignment, Lateral decubitus position

## Abstract

Introduction: Insertion of gamma nail with the patient in lateral decubitus position have the advantages of easier access to the entry point, easier fracture reduction and easier implant positioning. Our study described the incidence of femoral angular and rotational deformity following gamma nail insertion in lateral decubitus position.

*Methods*: In a prospective clinical case series, 31 patients (26 males and 5 females; the average age of 42.6 years) with 31 proximal femoral shaft fractures that were treated with gamma IMN were included in our study. Postoperatively, computerized tomography scans of the pelvis and both knees (injured and uninjured sides) were examined to measure anteversion angles on both sides. A scout film of the pelvis and upper both femurs was taken to compare the neck shaft angles on both sides.

*Results*: No angular malalignment was detected in our series; the mean angular malalignment angle was 1.6 ± 1.5°. There was a high incidence of true rotational malalignment of ≥10° in 16 out of 31 patients (51.6%); most of them were external rotational malalignment. Younger age group (≤40 years) had significantly more incidence of rotational malalignment (≥10°) than older age group (>40 years) (*P*-value 0.019).

*Discussion*: Gamma nail fixation in lateral decubitus position without the fracture table gives an accurate and easier access to the entry point, good implant positioning with no or minimal angular malalignment (varus −valgus) but poses high incidence of true rotational malalignment. Great care and awareness of rotation should be exercised during fixing proximal femoral fractures in lateral decubitus position.

## Introduction

The use of gamma nail (GN) is recommended in the treatment of unstable trochanteric and subtrochanteric fractures. However, there are several reported complications of gamma nail, including fracture of the femoral shaft (17%), failure of fixation (7%), and complications of distal locking (10%) [1–5].

Rotational deformity after locked proximal nail is the most common form of malunion with reported incidence in more than 40% of cases [6–10].

Using the lateral decubitus position in treating intertrochanteric fractures showed a satisfactory clinical outcome and a lower radiological complication rate as compared to the supine position. de Oliveira et al. [11], recommended insertion of cephalomedullary nails in peritrochanteric fractures in lateral decubitus position believing that lateral decubitus enables good fracture reduction and good implant positioning [11,12].

The aim of our study is to determine the incidence of femoral angular and rotational malalignment after GN insertion in the lateral decubitus position without a fracture table. To our knowledge, this is the first report of incidence of femoral rotational and angular malalignment after GN insertion in the lateral decubitus position without a fracture table.

## Patients and methods

This study was exempt according to the institutional review board of our institution. Informed consent was obtained from the patients following the guidelines set forth by our institution and by the Declaration of Helsinki and Good Clinical Practice. The consent included approval from the patient they will undergo a postoperative computerized tomography (CT).

In a prospective study, adult patients with unstable trochanteric (AO 31-A3.1) or subtrochanteric fractures treated with GN in the lateral decubitus position in the period from January 2014 to June 2015 were included. The exclusion criteria included stable trochanteric fractures i.e. with intact lesser trochanter, pathological fracture, and pregnant women. During this period 138 trochanteric fracture and 27 subtrochanteric fractures were admitted to emergency department of our hospital (tertiary care center). Sixty-one patients met the inclusion criteria but only thirty-one patients with 31 proximal femoral fractures accepted to participate in the study.

### Surgical technique

The patient was put under general or spinal anesthesia and positioned in lateral decubitus position on a radiolucent table with supporting pads. Before incision, anteroposterior and lateral images using the C-arm were taken to confirm correct positioning and to ensure a free pathway for the C-arm machine.

**Figure 1:**
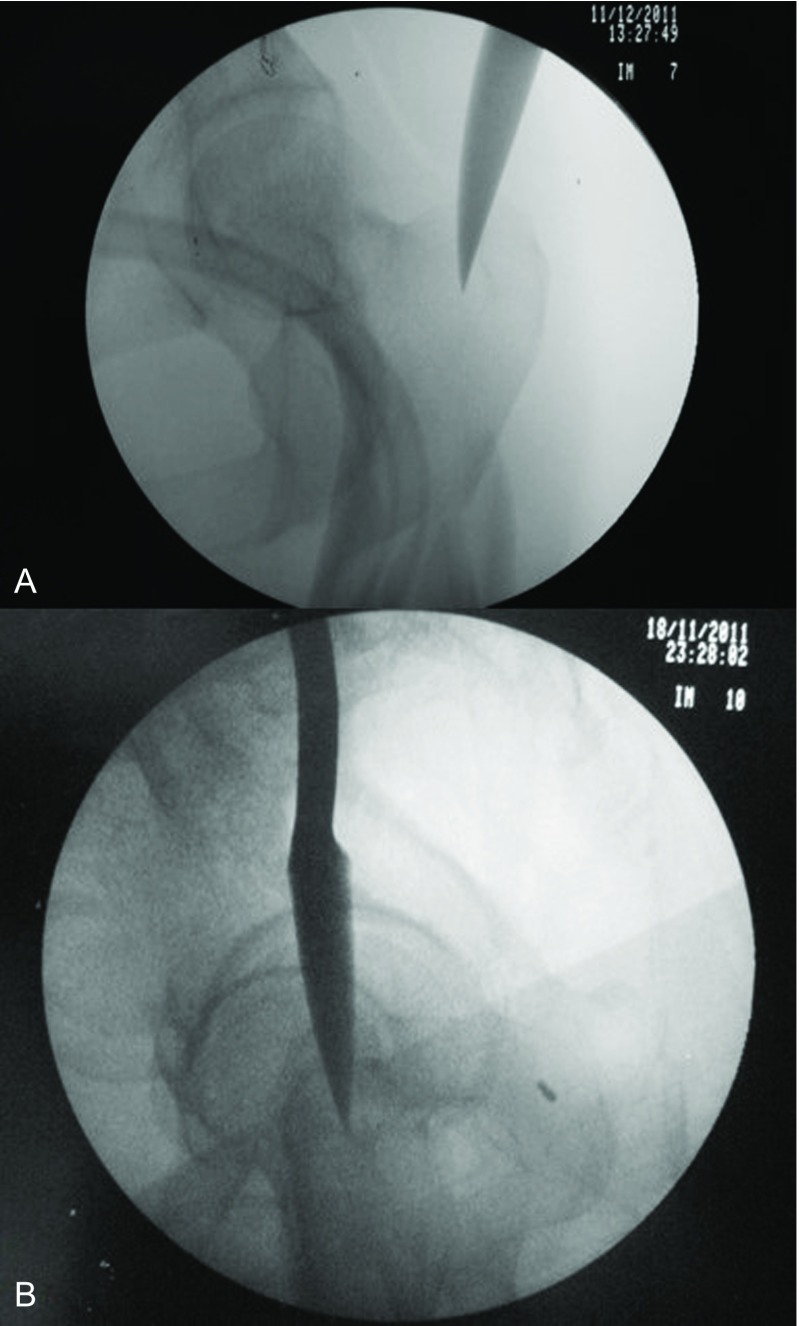
Anteroposterior and lateral fluoroscopic views of the entry point of gamma nail. In lateral view (b), the C-arm is positioned 10–15° off the perpendicular plane to the hip to overcome the overlap of the two hips.

**Figure 2:**
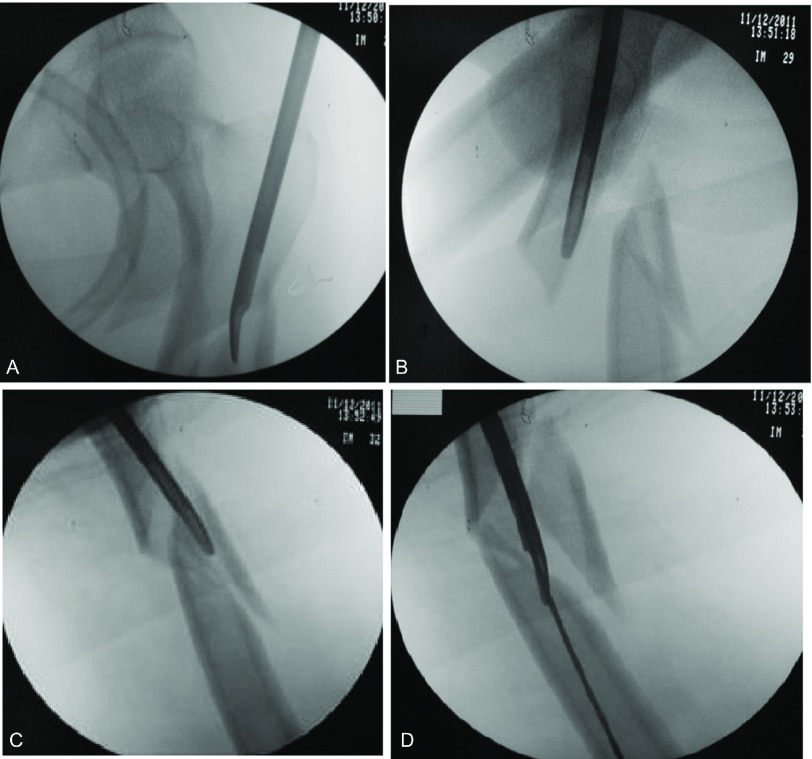
Using the internal reduction device to manipulate the proximal fragment aligning it with the distal fragment followed by passing the guide wire.

The ideal entry should be just medial to the tip of the greater trochanter in the coronal plane and at the junction of the anterior third and posterior two-thirds in the sagittal plane (Figure 1). Care was taken when interpreting the lateral view of the hip in the lateral decubitus position because of overlapping of the right and the left hip joints. Therefore, the C-arm is positioned at 10° to 15° deviation from the perpendicular plane to the hip joint to overcome this overlap. As a rule, the fracture has to be reduced before insertion of the GN. Manipulation of the fracture with traction and external or internal rotation with the help of folded draping sheet in the ipsilateral groin to reduce adduction of the proximal fragment. Internal reduction device can be also used to manipulate the proximal fragment to align it with the distal fragment and to help wire insertion (Figure 2). Joystick technique can be also used with the use of Schanz screws in the proximal and distal fragments. GN (Orthomed E Co®) was inserted by two of the authors (AM, EA) in all patients.

### Methods of evaluation

**Figure 3:**
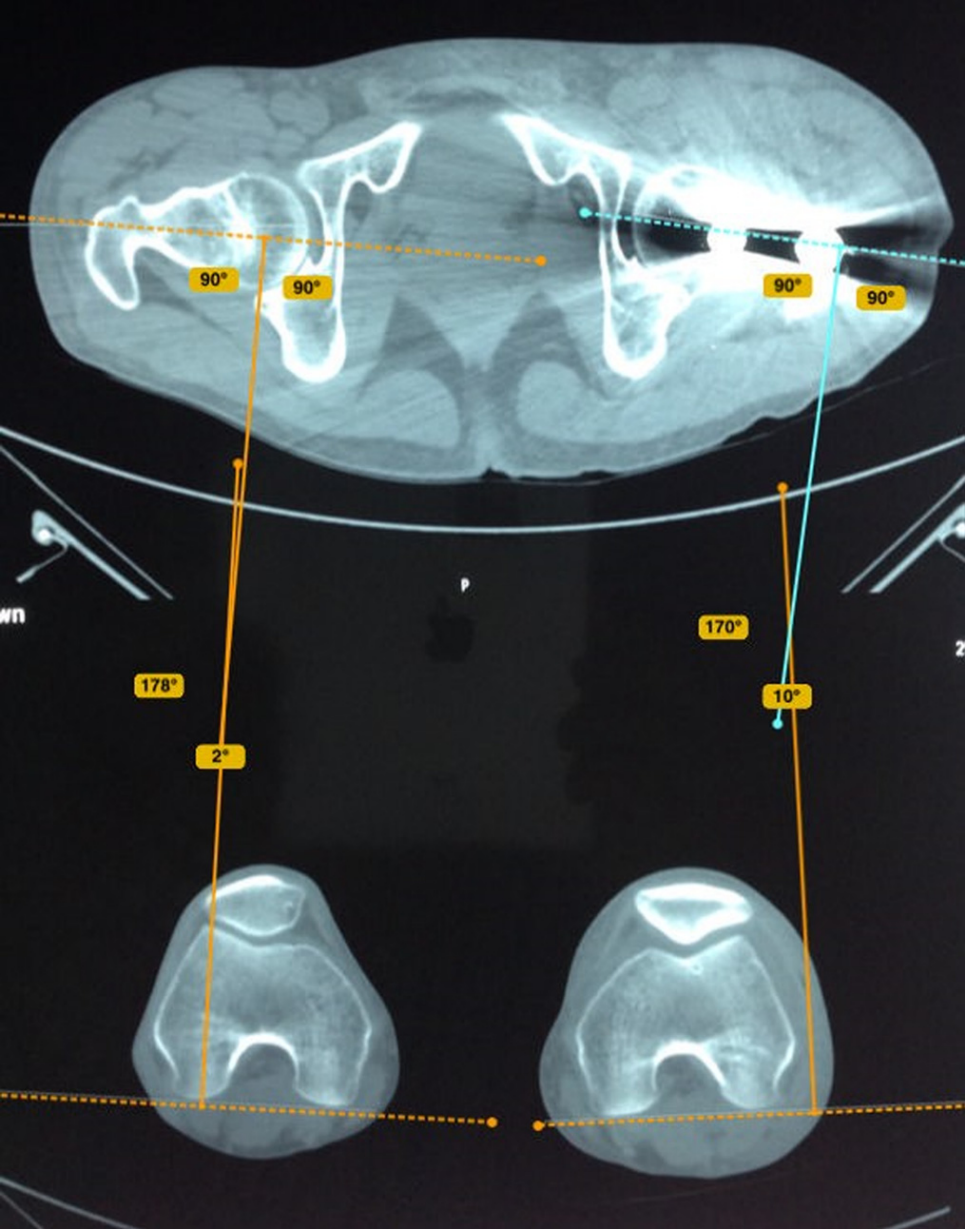
Measurement of anteversion angle on the fractured and healthy side by Bone Ninja application; a difference between both angles is expressed as rotational malalignment. On the injured left side, there is a difference in the rotation angle of 12° (increase in the angle means internal rotational malalignment of 10°−(−2°) = 12°).

**Figure 4:**
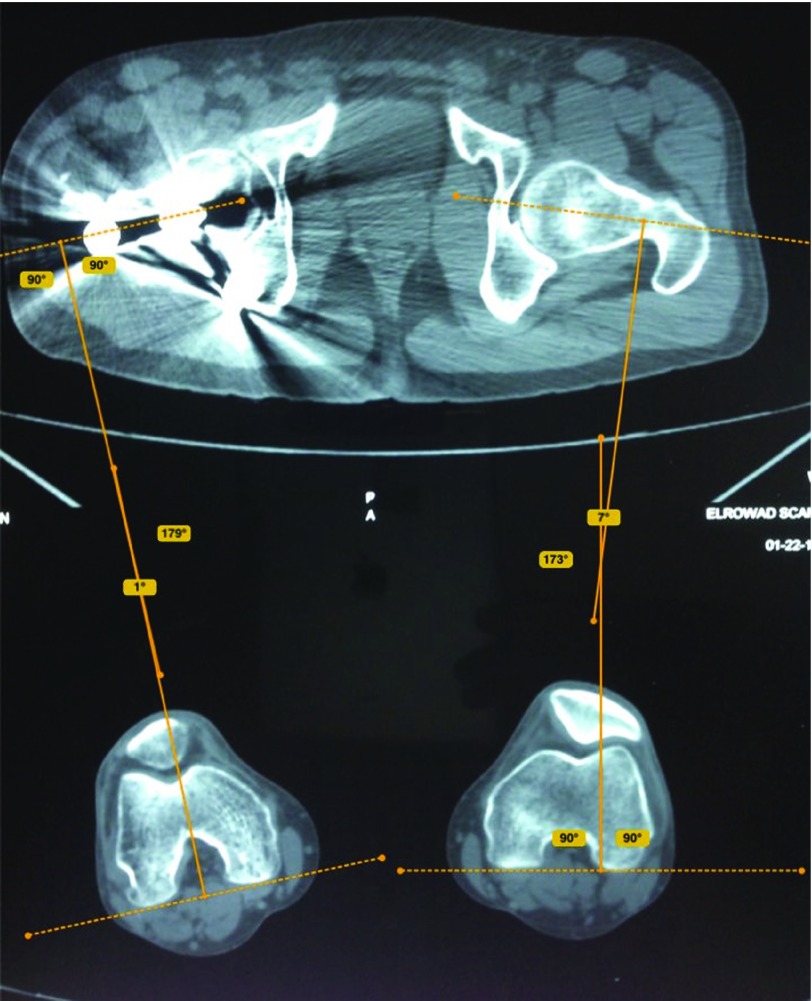
Measurement of anteversion angle on the fractured and healthy side by Bone Ninja application; a difference between both angles is expressed as rotational malalignment. On the injured right side, there is a difference in the rotation angle of 6° (decrease in the angle means external rotational malalignment of 7°−1° = 6°).

**Figure 5:**
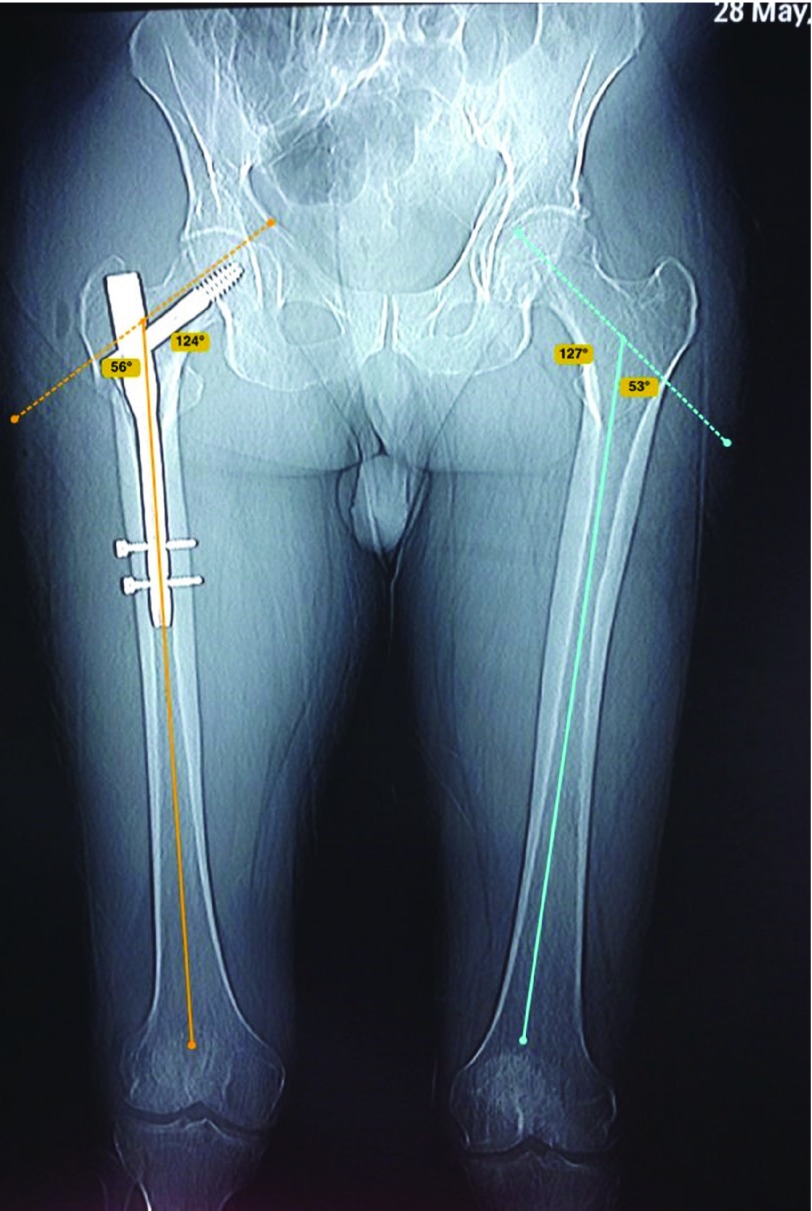
Measurement of neck-shaft angle on fractured and healthy side by Bone Ninja application.

Postoperatively, CT scans of the pelvis and both knees were examined to measure anteversion angles on both sides. A scout film of the pelvis and both femora was taken to compare neck shaft angles on both sides. Two authors (AH, FO) used Bone Ninja app (iPad) developed by Life Bridge health (version 4) from Apple Store to measure angular and rotational malalignment (Figures 3–5).

Rotational alignment was determined according to the method described by Jeanmart et al. [13] (Figures 3 and 4). The angle of anteversion is calculated as the angle between a line along the tangent of the posterior border of the femoral condyles and a line drawn through the femoral neck. Rotational malalignment was calculated by the angular difference between the fractured and unaffected side [13].

In external rotation deformity, the anteversion angle is decreased on the fractured side while in internal rotation deformity, the anteversion angle is increased. Patients with a difference in the torsion angle ≥10° were considered to have a true rotational malalignment [6,13].

The amount of angular malalignment was measured on the scout film of CT pelvis and both femora as the angle formed between two lines, one crossing the center of rotation of the femoral head and the center of the femoral neck, and the other along the long axis of the femoral shaft. (Figure 5). According to Paley et al. [14], the normal neck-shaft angle (NSA) is 129 ± 6.2° (range 124–136°). Patients with a difference in the NSA of ≥10° were considered to have a true angular malalignment.

### Statistical analysis

The data were tested for normality using the Anderson-Darling test and for homogeneity variances prior to further statistical analysis. Categorical variables were described by number and percent (*N*, %), where continuous variables described by mean and standard deviation (Mean, SD). Paired and unpaired *t*-test used to compare between continuous variables. A two-tailed *p* < 0.05 was considered statistically significant. All analyses were performed with the IBM SPSS 20.0 software.

## Results

Thirty-one patients with 31 proximal femoral fractures were available for study; 24 (77.4%) were subtrochanteric fractures and 7 (22.5%) were unstable trochanteric fractures. There were 26 male (83.9%) and 5 female (16.1%). The average age of patients was 42.6 ± 14.6 years (range 18–65 years). Younger age group (≤40 years) included 15 patients wheras older age group (>40 years) included 16 patients.

The mean rotational malalignment angle was 11.2 ± 8.2°. Sixteen of the 31 patients (51.6%) had a true rotational malalignment of ≥10°, fifteen of them (48.4%) had external rotation deformity and one patient (3.2%) had internal rotation deformity.

Younger age group (≤40 years) had significantly more incidence of true rotational malalignment (≥10°) than older age group (>40 years). Eleven (73.3%) out of 15 patients aged ≤40 years had true rotational malalignment of ≥10° while only 5 (31.3%) out of 16 patients aged >40 years had true rotational malalignment of ≥10° (*p*-value 0.019).

**Figure 6:**
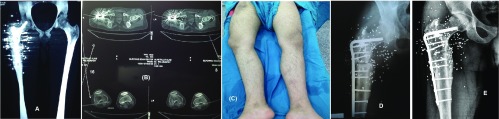
A 22-year old male patient with OGIII-A subtrochanteric femoral fracture due to gunshot injury managed with gamma nail, A) postoperative Scout film, B) postoperative CT scan with 19 degrees external rotation, C) external rotation deformity, D) X-ray after corrective osteotomy, E) 84 months follow up.

Four patients with rotational malalignment of ≥20° were readmitted to the theater within 2 to 4 days postoperative where the distal locking screw was removed, correction of rotational malalignment was done and re-locking done again. Other 12 patients were followed up closely; 5 of them with rotational malalignment of 10–14° doing well and no further interference was needed. Based on clinical finding such as limitation of internal rotation of the hip and ex-toeing gait, decision for corrective osteotomy was taken for 7 patients with rotational malalignment of 15–19° after an average of 12 months (range 6–18 months) after the index operation, but only three of them accepted a second surgery while the remaining patients refused to undergo second surgery (Figure 6).

We had no angular malalignment in our series. The mean angular malalignment angle was 1.6 ± 1.5° (range 0–4°), on the healthy side was 126.3 ± 3.3°, and on the fractured side was 126.9 ± 3.4°.

## Discussion

Complications of GN include fracture of the femoral shaft (17%), failure of fixation (7%), and complications of distal locking (10%) [5].

Torsional differences of less than 10° are considered variations of normal, while rotational malalignment ≥10° can cause hip and knee pain and restrict demanding activities like climbing stairs, running, and sports [9].

Alteration of joint loadings and biomechanics may eventually end up with degenerative arthritis of the hip and knee. Patients with a bigger degree of deformity >15° and those with external rotational deformity have significantly more symptoms than others [13,15–17].

CT is considered highly accurate in the measurement of femoral torsion. The inaccurate clinical assessment was the reason of very low incidence of rotational malalignment after femoral IM nailing described in early studies. It was 0.0% in the studies of Johnson et al. [18] and Kempf et al. [19] and 7% in the study of Wiss et al. [20]. While studies using CT measurement after antegrade femoral nailing reported higher incidence. It was 55% in the study of Jaarsma et al. [16], 42.7% in the study of Bråten et al. [21], and 40% in the study of Sennerich et al. [22]. None of these studies used the lateral position for femoral nailing.

Most of our cases with rotational malalignment occurred in our first cases when we shift the patient positioning from the supine position to the lateral position. We put folded draping in the groin only to reduce adduction of the proximal fragment. We did not put pads or draping between the knees. This caused the distal fragment to fall into adduction which is inevitably associated with external rotation. Ensuring that the tip of the greater trochanter, lateral epicondyle of the knee, and lateral malleolus are at the same horizontal level which is parallel to the floor is crucial before putting the distal locking screws to avoid rotational malalignment. To avoid femoral shortening, the two patellae should be at the same level in the same way as in total hip replacement in the lateral position. Routine application of computer navigation systems in the future may hold promise to decrease the high incidence of femoral rotational malalignment following closed IMN [23].

Younger age group (≤40 years) in our series had significantly more incidence of true rotational malalignment (≥10°) than older age group (>40 years) (*p*-value 0.019). This might be attributed to the fact that peritrochanteric fractures in young adults require a high energy trauma resulting in a highly comminuted and unstable fracture patterns. Multifragmentary comminuted fractures usually have difficulty in controlling rotation of fragments and this is possibly the cause behind higher incidence in younger patients. Difficulty to identify the surface landmarks in the muscular young patients is another possible explanation.

In this series, we had no true angular malalignment (≥10°). The tip of the greater trochanter is easily palpable in the lateral position that enables accurate entry point. Angular alignment is dependent mainly on the correct entry point, and this may be the reason why there were no cases of true angular malalignment in this study.

The major limitation of our study was the small number of patients. A larger number of patients is required to give solid conclusions and recommendations, however, the sample is representative of the problem. A further research study comparing rotational malalignment after GN of proximal femoral fractures in the lateral decubitus position with another group performed in the supine position is warranted.

## Conclusions

We concluded that insertion of GN with patients in the lateral decubitus position without a fracture table has the advantages of easier access to the entry point and resulted in good implant positioning in the femoral head and reduced complications associated with the use of fracture table. To avoid rotational malalignment, the tip of the greater trochanter, lateral epicondyle, and lateral malleolus should be at the same horizontal level before putting the distal locking screws to avoid rotational malalignment. Pads in the groin and between the knees will help to achieve this goal. The two patellae should be also checked for femoral length. Due to these advantages, it is advised to use the lateral decubitus position in fixing proximal femoral fractures with GN, but care should be taken to prevent or minimize rotational malalignment.

## Conflict of interest

The authors declare that there is no conflict of interests regarding the publication of this paper.
